# Frequentist and Bayesian Regression Approaches for Determining Risk Factors of Child Mortality in Ghana

**DOI:** 10.1155/2020/8168479

**Published:** 2020-10-06

**Authors:** Abdul-Karim Iddrisu, Kassim Tawiah, Francis Kwame Bukari, Williams Kumi

**Affiliations:** Department of Mathematics and Statistics, University of Energy and Natural Resources, School of Sciences, Ghana

## Abstract

**Background:**

Child mortality is a global health problem. The United Nations' 2018 report on levels and trends on child mortality indicated that under-five mortality is one of the major public health problems in Ghana with a rate of 60 deaths per 1000 live births. To further mitigate this problem, it is important to identify the drivers of under-five mortality in order to achieve the United Nations SDG Goal 3 target 2.

**Methods:**

In this study, we investigated the effects of some selected risk factors on child mortality using data from the 2014 Ghana Demographic Health Survey. We modelled the relationship between child mortality and the risk factors using a logistic regression model under the frequentist and Bayesian frameworks. We used the Metropolis-Hastings Algorithm to simulate parameter estimates from the posterior distributions, and statistical analyses were carried out using STATA version 14.1.

**Results:**

Results from the frequentist framework are in line with those from the Bayesian framework. The results showed an increased risk of death among children who were delivered through caesarean and reduced relative odds of death among children whose sizes are average or large at birth and whose mothers have formal education.

**Conclusions:**

There is a need for improved health facilities for better health-care for mothers and children. Education should, among other things, emphasise on the need for mothers to go for regular check-ups during antinatal and postnatal periods for improved mother and child health.

## 1. Background

One of the indicators that measure national development is child mortality [1, 2]. The world made remarkable progress in child survival in the past few decades, and millions of children have better survival chances than in 1990–5. It is known that 1 in 26 children died before reaching age five in 2018, compared to 1 in 11 in 1990. Moreover, progress in reducing child mortality has been accelerated in the 2000–2018 period compared with the 1990s, with the annual rate of reduction in the global under-five mortality rate increasing from 2.0 per cent in 1990–2000 to 3.8 per cent in 2000–2018. Despite the global progress in reducing child mortality over the past few decades, an estimated 5.3 million children under age five died in 2018—roughly half of those deaths occurred in sub-Saharan Africa [3].

The global under-five mortality rate declined by 59 per cent, from 93 deaths per 1000 live births in 1990 to 39 in 2018. Despite this considerable progress, improving child survival remains a matter of urgent concern. In 2018 alone, roughly 15,000 under-five deaths occurred every day, an intolerably high number of largely preventable child deaths [3, 4]. With the end of the MDG era, the international community agreed on a new framework—the Sustainable Development Goals (SDGs) where the target is to end preventable deaths of new-borns and children under-5 years of age. The goal is for all countries aiming to reduce under-five mortality to at least as low as 25 per 1000 live births. 120 Member States already met the SDG target on under-five mortality, and 21 countries are expected to meet the target by 2030, if the current trends continue [3].

Disparities exist in this reduction across countries. According to estimates from the Global Burden of Disease (GBD) 2017 SDG Collaborators, many countries are on track for achieving the target of at least 25 deaths per 1000 live births by 2030. However, about 31 countries/territories would need to achieve annual rates of decline from 2015 to 2030 that are two to ten times higher than what was recorded for 1990–2015 in order to achieve this goal [3, 5].

Despite the substantial decline in global under-five mortality, the rates remain high in sub-Saharan Africa where many countries like Ghana in the region failed to meet the Goal 4 of the Millennium Development Goals (MDGs) targets which aimed at a two-thirds reduction in the under-five mortality rate by 2015. In 2015, the under-five mortality rate in sub-Saharan Africa was 79 deaths per 1000 live births compared to the global rate of 41 deaths per 1000 live births in the same year [3, 4]. The under-five mortality rate in Ghana is still high with a rate of 60 deaths per 1000 live births in 2014 which fell short of the target set in the Ghana Under-five Child Health Policy 2007–2015 which targeted a reduction in under-five mortality to 40 deaths per 1000 live births by 2015 [6]. In Ghana, several national policies, strategies, and interventions with notable ones among them been the Child Health Policy 2007–2015, Community-based Health Planning and Services (CHPS) policy, and National Health Insurance (e.g., free maternal delivery services, free treatment of children aged below 18 years) were launched to improve and promote the health of Ghanaian children [6, 7]. Despite all these initiatives, under-five mortality in Ghana is still high in the country. A recent study conducted in 2016 among 46 African countries reported that Ghana is among the 8 countries that are making very little progress towards the reduction in under-five mortality [4, 8]. This calls for further examination of factors that may be militating against the expected reduction in the under-five mortality in the country. Aheto [4] modeled the relationship between under-five mortality and risk factors using univariate and multivariate logistic regression models (under the frequentist framework). In this paper, we modeled the relationship between child mortality and the risk factors using the logistic regression model under the frequentist [9–17] and Bayesian [18, 19] frameworks. We used the Metropolis-Hastings Algorithm to simulate parameter estimates from the posterior distributions, and statistical analyses were carried out using STATA version 14.1.

This paper is divided into 4 main sections. We have given the background of the study in Section 1. In Section 2, we introduce the study setting, source of data, response variable, risk factors, and statistical methods for the child mortality data.  Section 3 presents the results of the statistical analyses using the data.

## 2. Methods

In this section, we introduced the study setting and the source of data. We also introduced the response variable (child alive or dead) of interest as well as some potential predictors of the status of the response variable. This is followed by a discussion on the statistical approaches used in this study.

### 2.1. Study Setting and Data Source

This study is conducted in Ghana. The study used child mortality data on all the then ten regions in Ghana. The data were obtained from the Ghana Demographic and Health Survey for 2014. This study is cross-sectional, where the response variable of interest and its associated risk factors were measured at a single time point. In this study, we focused on individual child mortality data. The data used consist of 5868 children death records. Using these data, we categorized children into two groups; that is, those who are alive and those who are dead at the time of the survey. Also, we considered data on factors that are likely to determine whether a child is alive or dead. Ethical approval and consent to participate statements can be found on http://dhsprogram.com/What-We-Do/Protecting-the-Privacy-of-DHS-Survey-Respondents.cfm, approved by the ICF International Institutional Review Board (IRB). We will now introduce the outcome variable and the risk factors of child mortality.

### 2.2. Outcome Variable

In this study, the outcome variable of interest is child mortality status (which was coded 0 if a child is alive or 1 if a child is dead).

### 2.3. The Risk Factors or Predictors of Child Death Status

The status of the response variable, introduced in the previous section, depends on certain risk factors (also called predictors). These risk factors determine the status of the response variable. The risk factors used in this study are presented in Table 1.

The risk factors are gender (which was coded 0 if a child is female or 1 if a child is a male), where the child was delivered (which was coded 0 if a child was delivered at private and other health facilities or 1 if government health facility), caesarean section (CS) (which was coded 0 if a child was delivered normal or 1 if a child was delivered through CS), size of a child at birth (coded 0 if small, 1 average, or 2 if large), wealth index (was coded 0 if poor, 1 if average, or 2 if rich), geographical location (was coded 0 if rural or 1 if urban), and mother's educational status (was coded 0 if no formal education or 1 if formal education). Table 1 presents the percentage distribution of the background characteristics.

The percentage distributions of the variables in Table 1 showed that approximately 5% of the children died. There is a higher proportion (52%) of the male children with approximately 63% of the children delivered at government health facilities, and 10% of the children were delivered through caesarean section. Approximately 50% of the children were large in size at birth and 33% were average in size at birth, and a large proportion (54%) of children were from poor income homes. The majority (60%) of the children live in rural areas with more than 65% of the mothers having a formal education.

### 2.4. Statistical Analysis

First, we used the chi-Square test statistic [20–23] to investigate whether there is a significant association between the response (death status) and the predictors shown in Table 1. In our further analyses, we used the logistic regression [9–17] under the frequentist framework, to establish the relationship and to estimate the effects of the predictor variables on child mortality (death status). We then considered the logistic regression model under the Bayesian framework [18, 19]. We compared the results obtained under the frequentist logistic regression with that of the Bayesian framework. The purpose of this comparison is to check the robustness of conclusions under the frequentist framework to the formulations under the Bayesian framework.

#### 2.4.1. The Frequentist Logistic Regression Model

The general form of a logistic regression model [9, 10, 15–17, 24] can be written as

(1) $$\begin{array}{c} \text{logit}\left[\mathrm{Pr}\left(y_{i}\;|\;X,\beta \right)\right]=\text{logit}\left(p\right)=\log\left(\frac{p}{1-p}\right)=\beta_{0}+\beta_{1}X_{1}+\beta_{2}X_{2}+\cdots +\beta_{p}X_{p}, \end{array}$$

where  *X*_1_, ⋯, *X*_*p*_ are the predictors of child mortality, *β*_1_, ⋯*β*_*p*_ are the coefficients of regression, and *β*_0_ is the intercept. The coefficients of regression represent the magnitude and direction of the effects of **X** design matrix of the predictors on the dichotomous response variable  *y*_*i*_ and  **β**  is a vector of the regression coefficients. The *p*  in equation (1) represents the probability that a child has and  (*p*/1 − *p*) is the odds of death among children who are exposed to the predictors compared with those children who are not exposed to the same predictors. This implies that **β** is the log odds ratio of the death among children who are exposed to the predictors relative to those who are not exposed to the predictors [11]. This statistical method was implemented to the child mortality data using STATA version 14.1 software [25–28] for the estimation of the regression coefficients.

#### 2.4.2. Bayesian Regression Model

The Bayesian inference for logistic analyses is conducted the same way as the usual pattern for all Bayesian analyses. That is, we need to specify the likelihood function for the data and prior distribution for the parameters in the proposed regression model. Thereafter, we multiply the data likelihood function and the prior distribution for the parameter estimates to derive the posterior density, from which all statistical inferences are drawn.

The probability of success or death among the children varies from one child to another, depending on their risk factors. Hence, the likelihood function for the child mortality data follows a binomial distribution. Let *y* = (*y*_1_, *y*_2_,  ⋯, *y*_*n*_) be *n* independent binomial or binary random variables with the probability mass function defined as

(2) $$\begin{array}{c} f\left(y_{i}\;|\;\theta_{i}\right)=\left(\begin{array}{c} m_{i}\\ y_{i} \end{array}\right){\theta_{i}}^{y_{i}}{\left(1-\theta_{i}\right)}^{m_{i}-y_{i}},y_{i}=0,1,\cdots ,m_{i},m_{i}\geq 1,i=1,2,\cdots ,n, \end{array}$$

with *θ*_*i*_ = *F*(**X**′**β**), *F* is a cumulative distribution function, **X**′ is a *p*-dimensional covariates, and **β** is the vector of unknown regression coefficients, and *m*_*i*_ is the number of observations for the *i*th child. The logit link function was obtained by setting

(3) $$\begin{array}{c} F^{-1}\left(\theta_{i}\right)=\log\left(\frac{\theta_{i}}{1-\theta_{i}}\right)=X^{\prime }\beta , \end{array}$$

It follows from equation (2) that the likelihood function of binomial data can be defined as

(4) $$\begin{array}{c} f\left(y_{i}\;|\;\beta \right)=\overset{n}{\underset{i=1}{\prod }}\left(\begin{array}{c} m_{i}\\ y_{i} \end{array}\right){\theta_{i}}^{y_{i}}{\left(1-\theta_{i}\right)}^{m_{i}-y_{i}}. \end{array}$$

From expression (3), we can show that

(5) $$\begin{array}{c} \theta_{i}=\frac{\exp\left(x^{\prime }\beta =\beta_{0}+\beta_{1x_{i1}}+\beta_{2x_{i2}}+\cdots +\beta_{px_{ip}}\right)}{1+\exp\left(x^{\prime }\beta =\beta_{0}+\beta_{1x_{i1}}+\beta_{2x_{i2}}+\cdots +\beta_{px_{ip}}\right)}. \end{array}$$

It follows from expression (5) that the likelihood contribution from the *i*th child can be written as

(6) $$\begin{array}{c} f\left(y_{i}\;|\;\beta \right)=\overset{n}{\underset{i=1}{\prod }}\left(\begin{array}{c} m_{i}\\ y_{i} \end{array}\right){\left(\frac{\exp\left(x^{\prime }\beta =\;\beta_{0}+\beta_{1x_{i1}}+\beta_{2x_{i2}}+\cdots +\beta_{px_{ip}}\right)}{1+\exp\left(x^{\prime }\beta =\beta_{0}+\beta_{1x_{i1}}+\beta_{2x_{i2}}+\cdots +\beta_{px_{ip}}\right)}\right)}^{y_{i}}{\left(-\frac{\exp\left(x^{\prime }\beta =\beta_{0}+\beta_{1x_{i1}}+\beta_{2x_{i2}}+\cdots +\beta_{px_{ip}}\right)}{1+\exp\left(x^{\prime }\beta =\beta_{0}+\beta_{1x_{i1}}+\beta_{2x_{i2}}+\cdots +\beta_{px_{ip}}\right)}\right)}^{m_{i}-y_{i}}. \end{array}$$

In the expression (6), the unknown parameters are *β*_0_,  *β*_1_, ⋯,  *β*_*p*_. So, we need to specify prior distribution for these unknowns. We used the most common priors for logistic regression parameters, which are of the form: *β*_*j*_ ~ *N*(*μ*_*j*_,  *σ*^2^_*j*_). The distribution of the parameter estimates can be expressed explicitly as

(7) $$\begin{array}{c} f\left(\beta_{j}\right)=\frac{1}{\sqrt{2\pi \sigma_{j}}}\exp\left[-\frac{1}{2}{\left(\frac{\beta_{j}-\mu_{j}}{\sigma_{j}}\right)}^{2}\right] \end{array}$$

The most common choice for *μ*_*j*_  is zero, and *σ*_*j*_  is usually chosen to be large enough to be considered as noninformative.

As stated earlier, the posterior distribution is obtained by multiplying the prior distribution over all parameters by the full likelihood function as

(8) $$\begin{array}{c} f\left(\beta \;|\;y_{i},x_{ip},\right)=f\left(y_{i}\;|\;\beta \right)\times f\left(\beta \right). \end{array}$$

It follows from expression (8) that

(9) $$\begin{array}{c} f\left(\beta \;|\;y_{i},x_{ip}\right)=\overset{n}{\underset{i=1}{\prod }}\left(\begin{array}{c} m_{i}\\ y_{i} \end{array}\right)\left(\frac{\exp\left(x^{\prime }\beta =\;\beta_{0}+\;\beta_{1x_{i1}}+\beta_{2x_{i2}}+\cdots +\;\beta_{px_{ip}}\right)}{1+\exp\left(x^{\prime }\beta =\;\beta_{0}+\;\beta_{1x_{i1}}+\beta_{2x_{i2}}+\cdots +\;\beta_{px_{ip}}\right)}\right)\times^{y_{i}}{\left(-\frac{\exp\left(x^{\prime }\beta =\;\beta_{0}+\;\beta_{1x_{i1}}+\beta_{2x_{i2}}+\cdots +\;\beta_{px_{ip}}\right)}{1+\exp\left(x^{\prime }\beta =\;\beta_{0}+\;\beta_{1x_{i1}}+\beta_{2x_{i2}}+\cdots +\;\beta_{px_{ip}}\right)}\right)}^{m_{i}-y_{i}}\times \frac{1}{\sqrt{2\pi \sigma_{j}}}\exp\left[-\frac{1}{2}{\left(\frac{\beta_{j}-\mu_{j}}{\sigma_{j}}\right)}^{2}\right]. \end{array}$$

The above expression (9) has no closed-form expression. Even if it has a close form, we would have to carry out multiple integrations over the parameter estimates to obtain the marginal distribution for each coefficient. In this study, we use the Metropolis-Hastings Algorithm, with the bayesmh package in STATA, to simulate parameter estimates from the posterior distributions. Note that these parameter estimates are subject to Monte Carlo error, which is difficult to quantify. We have therefore chosen a very long run of which convergence was reached at 500000 after a burn-in period of 1000 and thinning of every 99th element of the chain for each model.

## 3. Results

### 3.1. Results from the Chi-Square Test Statistics

In Table 2, we presented the results of the chi-square test of association between the outcome variable (death status) and the potential predictors of death status. The purpose of this exercise is to identify variables that more likely to predict the status of the outcome variable.

The chi-square test statistic results showed that there is no significant association between the death status and gender: *χ*^2^ (df = 1, *n* = 5884) = 1.58, *p* value =0.209; place of delivery: *χ*^2^ (df = 1, *n* = 5884) = 3.67, *p* value =0.055; caesarean section: *χ*^2^ (df = 1, *n* = 5884) = 2.233, *p* value =0.127; wealth index: *χ*^2^ (df = 1, *n* = 5884) = 1.280, *p* value =0.526; or geographical location: *χ*^2^ (df = 1, *n* = 5884) = 0.02, *p* value =0.889 at 5% level of significance. The results also revealed a significant association between death status and child size at birth: *χ*^2^ (df = 1, *n* = 5884) = 26, *p* value =0.001; or mother education: *χ*^2^ (df = 1, *n* = 5884) = 5.62, *p* value =0.018. We observed that place of delivery: *χ*^2^ (df = 1, *n* = 5884) = 3.67, *p* value =0.055, and caesarean section: *χ*^2^ (df = 1, *n* = 5884) = 2.233, *p* value =0.127, are slightly significant (at 5% level of significance) and hence are likely determinants of death status when other risk factors are adjusted for.

Although the wealth index, geographical location, and gender variables were statistically insignificant (at 5% level of significance), they were included in the multiple logistic regression model to further assess their effects on child mortality and exclusion or inclusion in the logistic regression.

### 3.2. Results from the Logistic Regression Model

Our results showed that the wealth index (middle income, *p* value =0.743; rich, *p* value =0.610) and geographical location (*p* value =0.475) were highly statistically insignificant (at 5% level of significance), and hence, they were removed from the multiple logistic regression model. The best-fitting model has gender, delivery place, caesarean section, child size at birth, and mother's education variables included. These results are shown in the second column of Table 3. It can be observed that the unadjusted odds ratios (in the first column of Table 3) showed that gender, delivery place, and caesarean section are still statistically insignificant (at 5% level of significance). However, caesarean section becomes insignificant (at 5% level of significance) under the adjusted odds ratios (in the second column of Table 3).

For both the unadjusted and adjusted odds ratios, there is approximately 20% increased relative odds of death among male children compared to female children. However, these increases are not statistically significant (at 5% level of significance). The unadjusted odds showed that there is 21% reduced relative odds (OR = 0.792, 95%CI = 0.623, 1.006) of death among children who were delivered at government health facilities compared to private and other health facilities, whereas there is 20% reduced relative odds (OR = 0.804, 95%CI = 0.624, 1.036) of death among children who were delivered at government health facilities compared to private and other health facilities for the adjusted odds ratio. We observed these reductions are not statistically significant (at 5% level of significance).

The unadjusted odds showed that there is 1.3-folds increase in the relative odds (OR = 1.314, 95%CI = 0.924, 1.868) of death among children who were delivered through caesarean section. However, this increase is not statistically significant (at 5% level of significance). After adjusting for other risk factors of child mortality, there is a 1.4-fold increase in the relative odds (OR = 1.449, 95%CI = 1.005, 2.089) of death among children who were delivered through caesarean section compared to those who were born through normal delivery. This increase is statistically significant at 5% level of significance.

With the unadjusted odds ratios, we observed a 22% reduced relative odds (OR =0.780, 95% CI =0.600, 1.014) of death among children whose sizes are average at birth compared to those whose sizes are small at birth and 23% reduced relative odds (OR = 0.774, 95%CI = 0.610, 0.983) among children whose sizes are large at birth compared to those whose sizes are small at birth. When we adjusted for other risk factors of death, the relative odds (OR = 0.498, 95%CI = 0.362, 0.684) of death was reduced by 50% among children whose sizes are average at birth compared to those whose sizes are small at birth and 49% reduced relative odds (OR = 0.513, 95%CI = 0.384, 0.685) among children whose sizes are large at birth compared to those whose sizes are small at birth. The unadjusted odds ratio results showed that there was a 25% reduced relative odds (OR = 0.748, 95CI = 0.588, 0.952) of death among children whose mothers have formal education relative to those whose mothers have no formal education and 23% reduced relative odds (OR = 0.766, 95%CI = 0.596, 0.984) among children whose mothers have formal education relative to those whose mothers have no formal education when we adjust for other risk factors of child mortality. These reductions were found to be statistically significant.

### 3.3. Results from the Bayesian Logistic Regression Model

**Figure 1:**
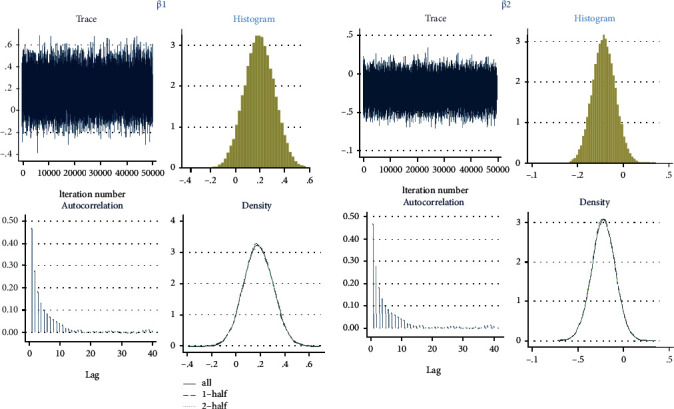
Gelman and Rubin's convergence diagnostics for gender and delivery place parameter estimates *β*_1_ and *β*_2_, respectively.

**Figure 2:**
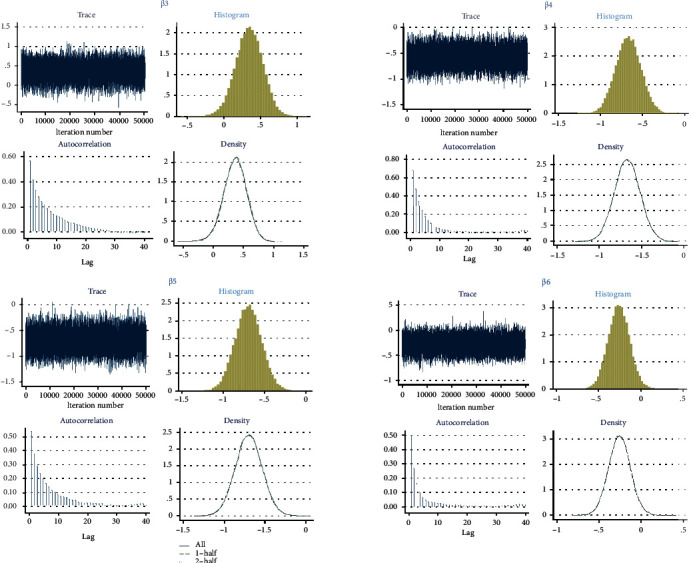
Gelman and Rubin's convergence diagnostics for caesarean section, large child size, average child size, and mother education parameter estimates *β*_3_,  *β*_4_, *β*_5_, and *β*_6_, respectively.

The results from fitting Bayesian logistic regression model to the child mortality data are shown in Table 4. Parameter estimation was carried out using the Markov Chain Monte Carlo (MCMC) via Metropolis-Hastings Algorithm. Convergence of the MCMC was reached at 500000 iteration after a burn-in period of 1000 sample and thinning of every 99th element of the chain. The Gelman and Rubin's convergence diagnostics for the parameters *β*_1_ (gender) and *β*_2_ (place of delivery) are presented in Figure 1. The Gelman and Rubin's convergence diagnostics of the parameter estimate *β*_3_ (caesarean section), *β*_4_ (large child size at birth), *β*_5_ (average child size at birth), and *β*_6_ (mother education) are presented in Figure 2. The traces, normality, and autocorrelation plots of these parameters showed that the Markov chain has converged.

The statistical inferences under the Bayesian paradigm are similar to those under the frequentist paradigm. Our results revealed that children who are males are at 21% increased relative odds (OR = 1.206, 95%CI = 0.950, 1.537) of death compared to females, and children who were delivered at government health facilities are at 20% reduced relative odds (OR = 0.805, 95%CI = 0.624, 1.037) of death compared to children who were delivered at private and other health facilities. We also found that children born through caesarean section have 1.4-fold increased relative odds (OR = 1.430, 95%CI = 0.981, 2.046) of death compared with those children who were born through normal delivery. We also found that large size children at birth have 49% reduced relative odds (OR = 0.513, 95%CI = 0.384, 0.689) of death compared with small size children at birth, and average size children at birth have 50% reduced relative odds (OR = 0.498, 95%CI = 0.363, 0.685) of death compared to small size children at birth. The results also showed that children whose mothers have formal education are at 23% reduced relative odds (OR = 0.766, 95%CI = 0.589, 0.986) of death compared to children whose mothers have no formal education.

Using the adjusted odds (under Tables 3 and 4), we observed that the size of a child at birth and mothers with formal education significantly reduce child mortality and caesarean section significantly increases child mortality.

## 4. Discussion

This paper investigated the effects of various risk factors on child's mortality in Ghana. The child's mortality status in this study was a dichotomous variable coded as 1 if a child was dead and 0 if a child was alive. The study used child mortality records data from the 2014 Ghana Demographic and Health Survey (2014GDHS). The data used for the analyses in this paper consist of 5884 complete cases [29–31] with 7 potential risk factors of child mortality. Analyses in this paper were carried out using the STATA software. We assessed the effects of the various risk factors on child mortality using a logistic regression model under the frequentist and the Bayesian frameworks. We built our regression models by first using a chi-square test statistic to determine potential predictors of child mortality.

We observed that 289 (4.91%) of the 5,884 children died. Risk factors such as caesarean section, size of the child at birth, and mother educational status were found to be significantly associated with child mortality. These study findings are in line with literature on child mortality in sub-Saharan Africa and developing countries. Child mortality is significantly higher among children who were delivered through caesarean section relative to those who were born through normal delivery. This is expected because literature [32–34] on child mortality and morbidity revealed that caesarean section is associated with an increased risk of child mortality. A possible explanation is that maintenance of body temperature, glycaemia, and pulmonary respiration in children born through caesarean section are delayed or affected [33]. Also, the development of a child's immune system is affected in children born through caesarean section [33].

There is a significant reduction in the risk of death among children whose sizes are average or large relative to those whose sizes are small at birth. This gives an indication that the diet of pregnant women must be balanced and health status regularly checked and improved for normal size or healthy children. A balanced diet for mothers will improve the nutritional status of the child or child health outcomes which will in turn lead to normal size child since studies [35–38] have shown that small child size is associated with increased risk of malnutrition.

A significant reduction of death among children whose mothers have education relative to those whose mothers have no education gives an indication that there is the need for improved maternal education which is likely to reduce mortality among children. This finding is in line with the findings from various authors [4, 39–41] on child mortality. Maternal education is expected to result in a reduction of child mortality because various authors [38, 39] revealed that improved maternal education is beneficial to mothers and their children as well as the society. Maternal education is beneficial to mothers and their children and hence is likely to reduce child mortality because mothers exposed to education are likely to utilize maternal health care services, such as antenatal care and postnatal care, for healthy mothers and children. Previous studies [37, 39, 41, 42] revealed that maternal education could offer mothers the opportunity to take part in making decisions that will improve the child health outcomes and utilization of maternal and child care services.

Our results also revealed that there is a reduction in the risk of child mortality among female children relative to male children. This study finding agrees with Aheto [4] finding, and the reason may be that female children are “more likely to develop early fetal lung maturity which will be protective of respiratory diseases unlike their male counterparts” [40, 43].

## 5. Conclusion

Although there is a slight difference in terms of estimates from the frequentist and Bayesian regression models, statistical inferences from the Bayesian logistic regression model agreed with that of the frequentist logistic regression model. Substandard children are more likely to die; there is higher child mortality among children who were delivered through caesarean section and low mortality among children whose mothers have formal education.

Based on the study findings, we recommend the government and health authorities concern to improve health facilities for better health-care for mothers and children. With such improvement of health facilities, there will be a more secured caesarean section, which is likely to significantly decrease the risk of child mortality due to caesarean section. Also, health authorities should intensify the education of mothers/families on the need or importance of using maternal health care services during pregnancy and after delivery. Education should, among other things, emphasize the need for mothers to go for regular check-ups during antinatal and postnatal periods for improved mother and child health. For standard or normal size children, we recommend that diet of mothers should be improved during antinatal care and postnatal care for better child's health which is likely to produce healthy (standard child with normal size) children which have the potential of reducing child mortality.

The first limitation of the study is that the authors were unable to adjust for potential risk factors that were not collected during the data collection process since the authors used secondary data. Second, the study could not utilize most risk factors because they have most of their values missing [29–31]. Third, because this study receives no funding, the authors were unable to raise funds for the collection of data on potential risk factors that were not considered during the survey or considered during the survey but could not be used because they have almost all their values missing.

This study considered only complete data (data without missing values) and hence could not take into account several other important determinants of child mortality because they have missing values or were not considered during the survey. Future studies should consider the use of primary data to allow for the collection of data on these variables and other important variables that were not considered during the survey. Also, future studies should employ sophisticated data management approaches for the imputation of missing data values to obtain a complete dataset [29] before statistical models are applied. Future studies can also consider models that account for missing values in the modelling process.

## Figures and Tables

**Table 1:** Percentage distribution of the response variable (death status) and the predictors.

Variable	*n* (%)
Death status	
No (alive)	5595 (95.09)
Yes (death)	289 (4.91)
Gender	
Female	2818 (47.89)
Male	3066 (52.11)
Delivery place	
Private and other health facilities	2172 (36.91)
Government	3712 (63.09)
Caesarean section	
No	5268 (89.53)
Yes	616 (10.47)
Child size at birth	
Small	1019 (17.32)
Average	1944 (33.04)
Large	2921 (49.64)
Wealth index	
Poor	3190 (54.21)
Middle	1083 (18.41)
Rich	1611 (27.38)
Geographical location	
Rural	3540 (60.16)
Urban	2344 (39.84)
Mother's education	
No formal education	2042 (34.70)
Formal education	3842 (65.30)

**Table 2:** Chi-square test of association between death status and predictors.

Variable	*χ* ^2^(df = 1, *n* = 5884), *p* value
Gender	1.58, 0.209
Delivery place	3.67, 0.055
Caesarean section	2.33, 0.127
Child size at birth	26, 0.001^∗∗^
Wealth index	1.28, 0.526
Geographical location	0.02, 0.889
Mother's education	5.62, 0.018^∗∗^

^∗∗^Variables with *p* value <0.05.

**Table 3:** Odds ratios (OR) and 95% confidence interval (95% CI) from frequentist logistic regression model.

Predictor	uaOR(95% CI)	aOR(95% CI)
Gender		
Female	1 (reference)	1 (reference)
Male	1.165 (0.918, 1.478)	1.202 (0.946, 1.527)
Delivery place		
Private and other health facilities	1 (reference)	1 (reference)
Government health facility	0.792 (0.623, 1.006)	0.804 (0.624, 1.036)
Caesarean section		
No	1 (reference)	1 (reference)
Yes	1.314 (0.924, 1.868)	1.449 (1.005, 2.089)^∗∗^
Child size at birth		
Small	1 (reference)	1 (reference)
Average	0.780 (0.600, 1.014)^∗∗^	0.498 (0.362,0.684)^∗∗^
Large	0.774 (0.610, 0.983)^∗∗^	0.513 (0.384, 0.685)^∗∗^
Mother's education		
No formal education	1 (reference)	1 (reference)
Formal education	0.748 (0.588, 0.952)^∗∗^	0.766 (0.596, 0.984)^∗∗^

^∗∗^Variables with *p* value <0.05.

**Table 4:** Parameter estimates, odds ratios (OR), and 95% confidence interval (95% CI) from the Bayesian logistic regression model.

Predictor	*β* (95% CI)	OR = exp(*β*) (95% CI)
Gender		
Female	1 (reference)	1 (reference)
Male	0.1876 (-0.052, 0.430)	1.206 (0.950, 1.537)
Delivery place		
Private and other health facilities	1 (reference)	1 (reference)
Government health facility	-0.217 (-0.471, 0.036)	0.805 (0.624, 1.037)
Caesarean section		
No	1 (reference)	1 (reference)
Yes	0.358 (-0.019, 0.716)	1.430 (1.002, 2.046)^∗∗^
Child size at birth		
Small	1 (reference)	1 (reference)
Average	-0.697 (-1.014, -0.378)^∗∗^	0. 498 (0.363, 0.685)^∗∗^
Large	-0.667 (-0.957, -0.372)^∗∗^	0.513 (0.384, 0.689)^∗∗^
Mother's education		
No formal education	1 (reference)	1 (reference)
Formal education	-0.267 (-0.514, -0.014)^∗∗^	0.766 (0.589, 0.986)^∗∗^

## Data Availability

The authors do not have the ability to make data available.
